# Sleep patterns and their associations with psychiatric symptoms among Chinese healthcare workers: a latent profile analysis

**DOI:** 10.3389/fpsyg.2024.1481580

**Published:** 2024-10-15

**Authors:** Yingjun Xiang, Shujuan Wei, Xiaoya Sun, Weiting Yang, Yaohui Han, Xuanzhen Wu

**Affiliations:** Shenzhen Futian Center for Chronic Disease Control, Shenzhen, Guangdong, China

**Keywords:** sleep patterns, latent profile analysis, psychiatric symptoms, healthcare workers, China

## Abstract

**Background:**

Healthcare workers often encounter inadequate sleep conditions. However, limited research has examined the underlying sleep patterns among healthcare workers. This study aimed to identify sleep patterns in healthcare workers, explore predictors associated with various sleep patterns, and investigate the relationship between sleep patterns and psychiatric symptoms.

**Methods:**

This cross-sectional study was conducted in Shenzhen, China, from April 2023 to June 2023. In total, data from 1,292 participants were included using a convenience sampling method. A latent profile analysis was conducted to identify sleep patterns based on the seven dimensions of the Pittsburgh Sleep Quality Index. Multinomial logistic regression analysis was conducted to investigate the influence of socio-demographic variables on each profile. A one-way ANOVA test was employed to examine the relationships between sleep patterns and psychiatric symptoms.

**Results:**

Three distinct profiles were identified: good sleepers (63.9%), inefficient sleepers (30.3%), and poor sleepers (5.7%). Multinomial logistic regression analysis indicated that gender and marital status were predictors of various sleep patterns. The ANOVA revealed significant differences in psychiatric symptoms scores among the three sleep patterns; poor sleepers exhibited the highest levels of mental distress.

**Conclusion:**

This study identified three distinct sleep patterns in healthcare workers and their significant associations with psychiatric symptoms. These findings contribute to the development of targeted intervention strategies aimed at improving sleep and reducing psychiatric symptoms among healthcare workers.

## 1 Introduction

Sleep is a crucial physiological activity for humans, significantly contributing to the elimination of metabolites, growth and development, and the regulation of immune function; it also functions as a restorative neurobehavioral state (Buysse, 2014).Healthcare workers are susceptible to sleep disorders due to shift work and occupational stress, which significantly impacts the quality of healthcare services (Jaradat et al., 2020; Johnson et al., 2014; Sun et al., 2022).Numerous studies have indicated that the prevalence of sleep disorders among medical personnel ranges from 38.9 to 46.1% (Marvaldi et al., 2021; Pappa et al., 2020; Valente et al., 2019). Consequently, there is a need for further research and a comprehensive understanding of the sleep status and patterns of healthcare workers to furnish evidence for enhancing sleep quality.

Sleep patterns are defined as an individual’s sleep behaviors and habits over a specific period, encompassing the time of falling asleep and awakening, sleep duration, the regularity of sleep (e.g., circadian rhythms), and its quality (Kryger et al., 2011). Measurement of sleep patterns includes objective measures (e.g., polysomnography, activity monitors), sleep logs, and self-report questionnaires (e.g., Pittsburgh Sleep Quality Index (PSQI) and self-administered questionnaires). Polysomnography (PSG) is a comprehensive sleep monitoring technique that provides a detailed assessment of an individual’s sleep status by recording multiple physiological parameters. These parameters typically consist of electroencephalogram (EEG), eye movements (EOG), electromyogram (EMG), electrocardiogram (ECG), respiratory airflow, oxygen saturation, and other physiological data. PSG is regarded as the gold standard for the diagnosis of sleep disorders and the conduct of sleep research (Iber et al., 2007). In recent years, as technology has evolved, an increasing number of wearable devices have been utilized to measure sleep patterns and quality. In a study conducted in Spain, researchers assessed sleep patterns by utilizing the ActiGraph GT9X Link and Fitbit Charge HR worn on the non-dominant wrists of elementary school children, while parents recorded the times of going to bed and waking up (Brazendale et al., 2019). Sleep log methods are simple and cost-effective ways to track and assess sleep; however, because they rely on subjective self-ratings, they can often be inaccurate and incomplete. A Canadian cross-sectional study utilized a sleep log where stroke patients reported experiencing difficulty staying awake most or all of the time to measure sleep disturbances (Jeffers et al., 2023). Standardized self-administered questionnaires are also commonly used to measure sleep patterns. In a study involving Chinese patients with schizophrenia, sleep patterns were categorized into short, medium, and long sleep based on patients’ self-reported expected sleep duration and actual total sleep duration (Hou et al., 2017).

Sleep patterns can be characterized by various dimensions, including sleep latency, sleep duration, insomnia medication use, and daytime dysfunction. Sleep patterns evolve in various ways throughout the lifespan, leading to declines in both the quantity and quality of sleep. A study found that 9.3% of younger individuals reported falling asleep later, whereas 22.5% of older individuals reported less efficient or poorer quality sleep (Gadie et al., 2017). Significant gender differences in sleep patterns have been also observed, with women more likely to report disturbed sleep onset and men more likely to report nocturnal awakenings (Middelkoop et al., 1996). Furthermore, a British cohort study indicated that sleep proportion (the ratio of sleep duration to time in bed) is a useful indicator of sleep patterns in the general population (Leng et al., 2014). Nevertheless, limited research has focused on the underlying sleep patterns of healthcare workers. Therefore, there is a critical need for studies focused on healthcare workers, which are essential for developing targeted intervention strategies tailored to various sleep patterns to enhance sleep quality.

Established studies have demonstrated a potential causal relationship between sleep disorders and psychiatric symptoms in healthcare workers (Dong et al., 2017; Kalmbach et al., 2017; Liu et al., 2022; Sørengaard et al., 2019). A meta-analysis revealed that healthcare workers with sleep disorders faced a 3.74-fold increased risk of psychiatric symptoms compared to those without sleep disorders (Liu et al., 2022).These results may stem from the vital functions of sleep in the human body, which include maintaining consciousness and cognitive function, regulating biorhythms, enhancing immune defenses, and alleviating stress (Ding et al., 2022; Dinges et al., 1995). Therefore, understanding the complexities of sleep patterns is essential for elucidating their impact on psychiatric symptoms and overall wellbeing. Latent profile analysis (LPA) has been widely utilized in psychological and humanities research to identify individuals with varying personal attributes (Hensel, 2020).This approach can assist in exploring the sleep patterns of healthcare workers, further delineate distinct subgroups with unique sleep patterns, and facilitate the development of targeted intervention strategies for sleep management. The aims of this study were: (1) to identify the sleep patterns of healthcare workers through LPA, indexed by the seven sleep dimensions of the Pittsburgh Sleep Quality Index (PSQI); (2) to understand the predictors of varying sleep patterns in healthcare workers; and (3) to examine the relationship between sleep patterns and the psychiatric symptoms among healthcare workers.

## 2 Methods

### 2.1 Study design and participants

A cross-sectional study was conducted in Shenzhen, China, from April 2023 to June 2023. All seven public hospitals in the Futian District of Shenzhen were invited to participate. Researchers initially contacted the personnel departments of the seven public hospitals and obtained their consent to conduct the survey. Subsequently, with assistance from the personnel departments, healthcare workers (including all doctors and nurses) from all departments who met the inclusion criteria were requested to complete the questionnaire. The eligibility criteria for participants were as follows: (a) employed in hospitals as healthcare workers for 6 months or more, (b) aged 18–65 years, and (c) mentally competent and willing to participate in the study. The exclusion criteria included individuals: (a) who had retired or were subsequently rehired in hospitals, and (b) who were diagnosed with a mental disorder.

A total of 1,352 healthcare workers participated in this study. Participants were excluded from the analysis if they completed less than 80% of the two target questionnaires, finished the survey in under 60 s, were suspected of being non-responders (i.e., providing identical responses), or missed one or more questions. Ultimately, data from 1,292 participants were included in the final statistical analysis.

### 2.2 Procedure

All assessments were conducted within the Mental Health Assessment System of the Futian District in Shenzhen. Healthcare workers accessed the system by scanning a QR code and subsequently answered the questions independently. The questionnaire included socio-demographic information, sleep status, and psychiatric symptoms. The questionnaire was not anonymous allowing for the identification and tracking of healthcare workers for subsequent studies. The voluntary nature of participation and confidentiality of information were explicitly stated at the beginning of the questionnaire. This study was conducted in accordance with the guidelines of the Declaration of Helsinki (World Medical Association Declaration of Helsinki, 2002) and received approval from the Ethics Committee of the Shenzhen Futian Center for Chronic Disease Control (No. 2023003).

### 2.3 Measures

#### 2.3.1 Socio-demographic factors

A self-designed questionnaire was utilized to collect socio-demographic variables, including gender, age, marital status, educational level, years of work in hospitals, professional title, and categories of hospitals. Age was assessed in years and subsequently stratified into four categories: 18–34 years, 35–44 years, 45–55 years, and 55 years and older. The types of hospitals were categorized as ‘tertiary,’ ‘secondary,’ and ‘primary’ hospitals. In China, hospitals are classified into three grades based on size, research focus, human resources, technical strength, and medical equipment. Table 1 provides additional details regarding the classification of socio-demographic variables for this study.

**Table 1:** Characteristics of Chinese healthcare workers (*N* = 1,292).

Variables	*N* (%)
Gender	
Male	395 (30.6)
Female	897 (69.4)
Age, years	
18–34	143 (11.1)
35–44	669 (51.8)
45–55	392 (30.3)
>55	88 (6.8)
Marital status	
Unmarried	76 (5.9)
Married	1,157 (89.6)
Other	59 (4.6)
Education level	
Junior college or below	65 (5.0)
Undergraduate	885 (68.5)
Postgraduate or above	342 (26.5)
Working years in hospital	
<5	18 (1.4)
5–14	368 (28.5)
15–24	518 (40.1)
>25	388 (30.0)
Professional title	
Junior	123 (9.5)
Intermediate	676 (52.3)
Senior	463 (35.8)
Other	30 (2.3)
Categories of hospital	
Tertiary hospital	765 (59.2)
Secondary hospital	342 (26.5)
Primary hospital	185 (14.3)
Overall sleep (PSQI, cutoff of 7)	
Good	886 (68.6)
Poor	406 (31.4)
Overall psychiatric symptoms (scl-90, cutoff of 160)	
Good	1,199 (92.8)
Poor	93 (7.2)

#### 2.3.2 PSQI

The 19-item self-reported Pittsburgh Sleep Quality Index (PSQI) was used to evaluate overall sleep across seven dimensions: subjective sleep quality, sleep latency, sleep duration, sleep efficiency, sleep disturbances, sleep medication use, and daytime dysfunction. The total score ranged from 0 to 21, with each component scored between 0 and 3. Higher scores indicated poorer sleep quality, with a global score exceeding 7 suggesting poor overall sleep quality (Liu and Hu, 1996). The Chinese version of the PSQI has been translated and validated, and it has been widely used in China, demonstrating satisfactory reliability and validity (Zhang et al., 2017; Zhang et al., 2021). The Cronbach’s alpha coefficient for this study was 0.867.

#### 2.3.3 SCL-90

Psychiatric symptoms was assessed using the Symptom Checklist-90 (SCL-90), the most commonly employed screening scale for evaluating mental and psychological disorders (Albert et al., 2022; Ignatyev et al., 2016). The SCL-90 comprises 90 items across 10 domains: somatization, obsessive-compulsiveness, interpersonal sensitivity, depression, anxiety, hostility, phobic anxiety, paranoid ideation, psychoticism, and sleep difficulties. Each item is scored on a scale of 1 to 5, resulting in a total score ranging from 90 to 450. Higher scores indicate more severe psychiatric symptoms. A total score of ≥160 on the SCL-90was considered indicative of “poor psychiatric status” (Du et al., 2020).The Chinese version of PSQI has been translated and validated, and it has been widely used as a widely measure of psychiatric symptoms in China (Xie et al., 2021; Zhong et al., 2013). The Cronbach’s alpha coefficient for this study was 0.988.

#### 2.3.4 Statistical analysis

Descriptive statistics for socio-demographic factors, overall sleep, and psychiatric symptoms were reported as proportions. Since the scores of the seven dimensions of PSQI could be processed mathematically (e.g., averaging, summing, etc.), they were considered continuous variables in this study. Latent profile analysis allowed for the classification of individuals into different categories based on the characteristics of their responses to continuous variables. LPA was conducted to identify the sleep patterns of Chinese healthcare workers using seven continuous variables: subjective sleep quality, sleep latency, sleep duration, sleep efficiency, sleep disturbances, sleep medication use, and daytime dysfunction. To ensure comparability across items, the total scores of each subscale of PSQI were averaged. Various fit indicators, including the Akaike Information Criterion (AIC), Bayesian Information Criterion (BIC), and adjusted Bayesian Information Criterion (aBIC), were used to determine the optimal number of latent profiles. Lower AIC, BIC, and aBIC values indicated better model fit. The Lo–Mendell–Rubin (LMR) and bootstrap likelihood ratio test (BLRT) were used to compare models, with a significant *p*-value indicating a better fit for the k-class model. The entropy was assessed for classification precision, with higher values indicating more accurate categorization (ideally above 0.80) (Tein et al., 2013). Additionally, an extra class would be rejected if it included fewer than 50 cases or less than 5% of the total sample size (Weller et al., 2020).Following the identification of optimal sleep patterns, a one-way ANOVA was conducted to assess differences across sleep patterns concerning the seven sleep dimensions. Subsequently, multinomial logistic regression was performed to investigate the impact of socio-demographic variables on each identified profile. Lastly, the relationship between profile membership and psychiatric symptoms was evaluated using an ANOVA. Descriptive statistics, ANOVA, and multinomial logistic regression were performed using SPSS Version 27 (George and Mallery, 2018) while LPA was conducted with Mplus Version 8.3 (Muthén and Muthén, 2017).

## 3 Results

### 3.1 Test for common method bias

Due to the self-report data collection method from a single source, Harman’s single-factor test was conducted prior to data analysis. This involved conducting an exploratory factor analysis to identify any common method bias. The results indicated the presence of three factors with eigenvalues exceeding 1, which accounted for 67.6% of the variance. The variance explained by the first factor was 22.3%, significantly below the critical threshold of 40% (Podsakoff et al., 2003). Therefore, there is no significant common method bias present in this study.

### 3.2 Participant characteristics

The descriptive statistics for each study variable were shown in Table 1. Among the 1,292 participating healthcare workers, 69.4% were female, with an average age of 43.97 years (SD = 6.95 years; range: 25–64 years), and 89.6% were currently married. 95% held an undergraduate degree or higher. Additionally, 70.1% had worked in medical institutions for over 15 years. 90% held mid-level or higher professional titles, and 59.2% worked in tertiary hospital. Moreover, 31.4% rated their sleep status as poor, while 7.2% rated their psychiatric status as poor.

### 3.3 Latent profile analysis

Table 2 presented the model fit indices from the 1-class to the 5-class model. The AIC, BIC, and aBIC exhibited a general decrease with an increasing number of estimated models, while entropy consistently remained above 0.80. Based on the pLMR test, the 5-class model did not significantly improve the model fit compared to the 4-class model (*p* = 0.09). Compared to the 4-class model, the 3-class model exhibited a higher entropy value (0.999), with the smallest group comprising more than 5% of the total sample. Within the 3-class model, the average probability of correct assignment for each profile was 1.000, indicating minimal probabilities of misattribution. Consequently, the 3-class model demonstrated an overall good fit and was the most parsimonious and interpretable option.

**Table 2:** Fit indices for five models using latent profile analysis (*N* = 1,292).

Latent classes	AIC	BIC	aBIC	Entropy	pLMR	pBLRT	Proportions Min (%)
1	22430.762	22503.058	22458.587	–	–	–	
2	20230.910	20344.516	20274.633	1.000	<0.001	<0.001	32.5
**3**	**18709.666**	**18864.584**	**18769.289**	**0.999**	**<0.001**	**<0.001**	**5.7**
4	17720.815	17917.045	17796.338	0.899	0.0299	<0.001	3.4
5	16869.654	17107.195	16961.076	0.998	0.09	<0.001	5.7

Boldface indicates the selected model. AIC, Akaike Information Criterion; BIC, Bayesian Information Criterion; aBIC, adjusted BIC; pLMR, *p*-value for LoMendell-Rubin adjusted likelihood ratio test for K vs. K-1 profiles; pBLRT, *p*-value for Bootstrapped Likelihood Ratio Test.

**Figure 1:**
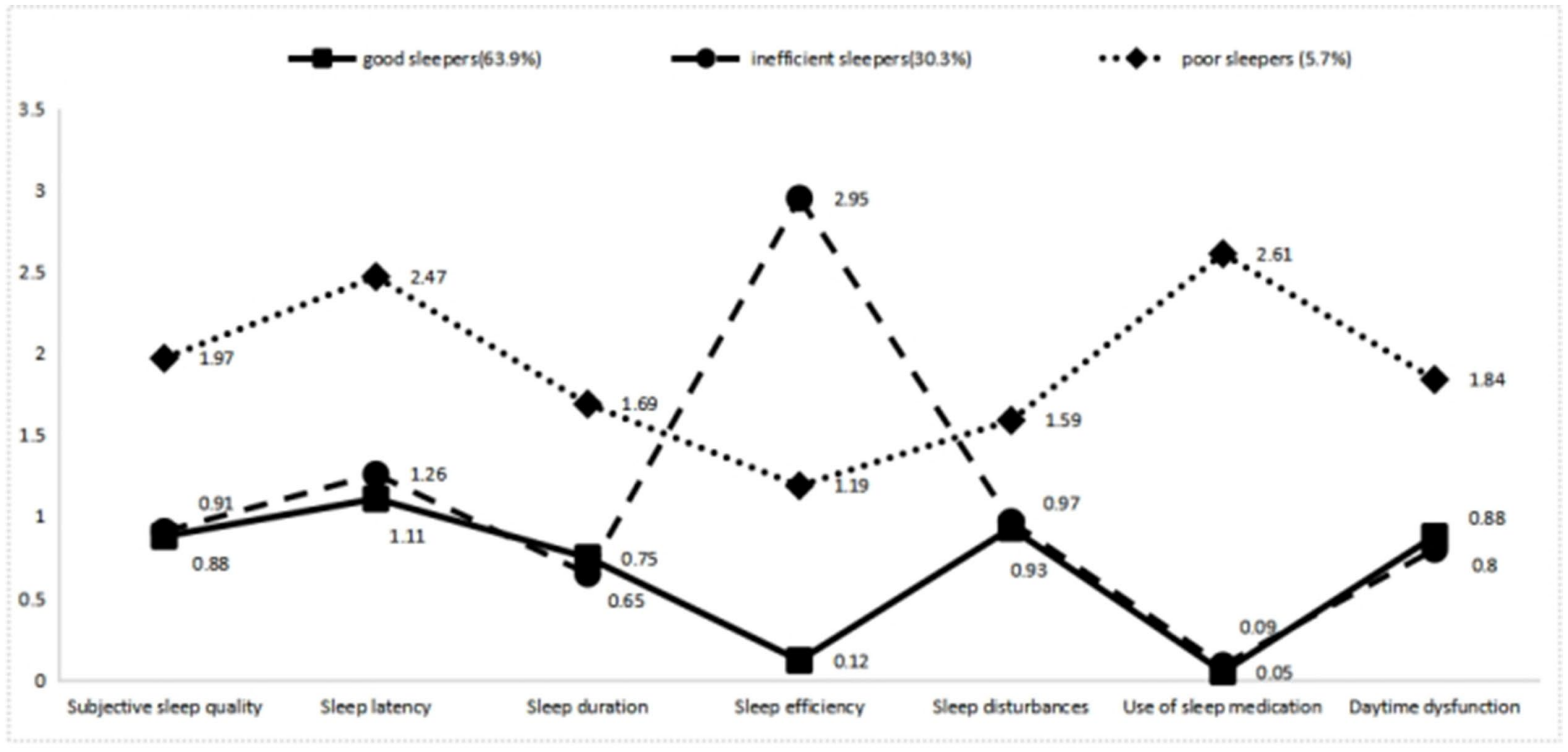
Profile of the sleep patterns in Chinese healthcare workers.

Figure 1 and Table 3 illustrated significant differences in the seven sleep dimensions among the three profiles (*p* < 0.001). In general, healthcare workers in Classes 1 and 2 exhibited lower scores in all sleep dimensions, except for sleep efficiency. Healthcare workers in Class 3 reported higher scores in all seven dimensions compared to those in Class 1 and Class 2. Healthcare workers in Class 2 demonstrated a significantly higher score in sleep efficiency compared to those in Classes 1 and 3. Consequently, the three identified sleep patterns in this study were categorized as follows: good sleepers (Class 1, 63.9%), inefficient sleepers (Class 2, 30.3%), and poor sleepers (Class 3, 5.7%).

**Table 3:** Differences in the seven sleep dimensions among the three profiles.

Variable	Class 1 (M ± SD)	Class 2 (M ± SD)	Class 3 (M ± SD)	*F* ^a^
Subjective sleep quality	0.88 ± 0.701	0.91 ± 0.674	1.97 ± 0.702	86.093^***^ (1 = 2 < 3)
Sleep latency	1.11 ± 0.98	1.26 ± 0.989	2.47 ± 0.798	66.624^***^ (1 = 2 < 3)
Sleep duration	0.75 ± 0.743	0.65 ± 0.798	1.69 ± 0.992	56.698^***^ (1 = 2 < 3)
Sleep efficiency	0.12 ± 0.331	2.95 ± 0.225	1.19 ± 1.341	5656.099^***^ (1 < 3 < 2)
Sleep disturbances	0.93 ± 0.555	0.97 ± 0.539	1.59 ± 0.81	47.024^***^ (1 = 2 < 3)
Sleep medication use	0.05 ± 0.22	0.09 ± 0.286	2.61 ± 0.492	3260^***^ (1 = 2 < 3)
Daytime dysfunction	0.88 ± 0.871	0.80 ± 0.878	1.84 ± 0.907	45.302^***^ (1 = 2 < 3)

Class1, good sleepers group; Class2, inefficient sleepers group; Class3, poor sleepers group. ^***^p < 0.001. ^a^ANOVA and *post-hoc* pairwise Bonferroni tests for the dimension indicators with a normal distribution.

### 3.4 Predictor of latent profile membership

Using “poor sleepers” as the reference group, a multinomial logistic regression analysis was conducted to examine the predictors of profile membership (Table 4). Compared to females, males exhibited higher odds of belonging to “good sleepers”(OR: 2.31, 95% CI: 1.222–4.366). Married healthcare workers, compared to others, showed a higher likelihood of members of the “inefficient sleepers” (OR: 3.797, 95% CI: 1.427–10.100).

**Table 4:** Multinomial logistic regression results predicting latent profile membership.

Variable	Good sleepers			Inefficient sleepers		
	β	OR (95%CI)	P	β	OR (95%CI)	P
Gender (ref: female)						
Male	**0.837**	**2.310 (1.222,4.366)**	**0.010**	0.021	1.021 (0.523,1.994)	0.951
Age (ref: >55)						
<35	0.341	1.406 (0.195,10.143)	0.735	−0.404	0.668 (0.088,5.084)	0.697
35–45	0.428	1.535 (0.387,6.089)	0.542	−0.346	0.707 (0.169,2.963)	0.636
46–55	−0.610	0.543 (0.176,1.667)	0.289	−1.073	0.342 (0.107,1.097)	0.071
Marital status (ref: other)						
Unmarried	0.786	2.195 (0.477,10.099)	0.313	0.957	2.604 (0.497,13.627)	0.257
Married	0.163	1.845 (0.797,4.271)	0.153	**1.334**	**3.797 (1.427,10.100)**	**0.008**
Education level (ref: Postgraduate or above)						
Junior college or below	−0.571	0.565 (0.195,1.641)	0.294	−0.813	0.444 (0.142,1.389)	0.163
Undergraduate	−0.294	0.745 (0.375,1.483)	0.403	−0.255	0.755 (0.380,1.580)	0.483
Working years in medical institution (ref: >25)						
<5	−0.226	0.797 (0.053,11.980)	0.870	−0.313	0.731 (0.044,12.296)	0.828
5–14	0.672	1.98 (0.566,6.766)	0.288	−0.898	2.455 (0.680,8.865)	0.170
15–24	0.065	1.067 (0.507,2.248)	0.864	0.090	1.094 (0.496,2.414)	0.824
Professional title (ref: other)						
Junior	0.574	1.776 (0.385,8.190)	0.462	1.346	3.841 (0.719,20.526)	0.116
Intermediate	0.445	1.561 (0.417,5.841)	0.508	0.861	2.367 (0.538,10.414)	0.255
Senior	0.679	1.972 (0.509,7.647)	0.326	0.869	2.385 (0.523,10.877)	0.262
Categories of medical institution (ref: primary hospital)						
Tertiary hospital	0.190	1.210 (0.602,2.430)	0.592	0.182	1.200 (0.579,2.487)	0.625
Secondary hospital	0.233	1.262 (0.580,2.745)	0.557	0.193	1.213 (0.539,2.733)	0.640

Base outcome: “poor sleepers.” OR, odds ratio; CI, confidence interval.Bold values indicate statistically significant predictors.

### 3.5 Comparisons of the different profiles by psychiatric symptoms

**Figure 2:**
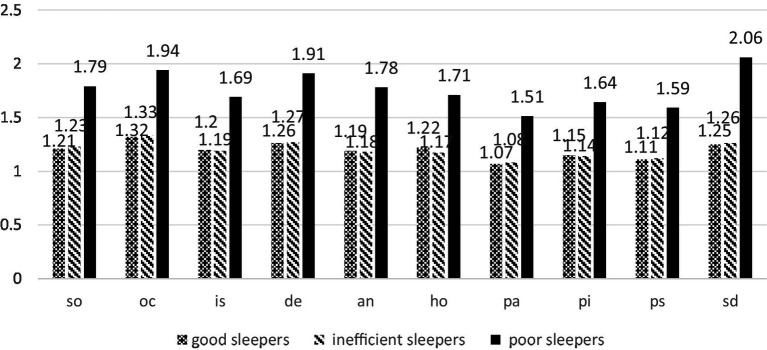
Results of the variance analyses conducted on the psychiatric symptoms; means of the three profiles are displayed. so, somatization; oc, obsessive-compulsive; is, interpersonal sensitivity; de, depression; an, anxiety; ho, hostility; pa, phobic anxiety; pi, paranoid ideation; ps, psychoticism; sd, sleep difficulties.

The one-way ANOVA (Table 5 and Figure 2) revealed a significant difference among latent profile groups for all dimensions of psychiatric symptoms (*F* = 44.597–119.139, *p* < 0.001). Poor sleepers exhibited higher scores of psychiatric symptoms scores compared to the other two groups (*p* < 0.001). Additionally, the differences between “good sleepers” and “inefficient sleepers” were not statistically significant, except for hostility.

**Table 5:** Comparisons of the different profiles by psychiatric symptoms (M ± SD).

Variables	Class 1	Class 2	Class 3	*F*	*P*	*Post-hoc*
Somatization	1.21 ± 0.36	1.23 ± 0.38	1.79 ± 0.97	63.834	<0.001	1 = 2 < 3
Obsessive-compulsive	1.32 ± 0.44	1.33 ± 0.47	1.94 ± 1.01	53.326	<0.001	1 = 2 < 3
Interpersonal sensitivity	1.20 ± 0.37	1.19 ± 0.40	1.69 ± 0.99	44.597	<0.001	1 = 2 < 3
Depression	1.26 ± 0.44	1.27 ± 0.47	1.91 ± 1.08	56.098	<0.001	1 = 2 < 3
Anxiety	1.19 ± 0.36	1.18 ± 0.35	1.78 ± 1.00	67.320	<0.001	1 = 2 < 3
Hostility	1.22 ± 0.39	1.17 ± 0.29	1.71 ± 0.98	53.781	<0.001	2 < 1 < 3
Phobic anxiety	1.07 ± 0.21	1.08 ± 0.24	1.51 ± 0.92	71.207	<0.001	1 = 2 < 3
Paranoid ideation	1.15 ± 0.32	1.14 ± 0.34	1.64 ± 0.40	54.870	<0.001	1 = 2 < 3
Psychoticism	1.11 ± 0.27	1.12 ± 0.28	1.59 ± 0.93	66.750	<0.001	1 = 2 < 3
Sleep difficulties	1.25 ± 0.38	1.26 ± 0.41	2.06 ± 0.92	119.139	<0.001	1 = 2 < 3

Class 1, good sleepers group; Class 2, inefficient sleepers group; Class 3, poor sleepers group. 1 = Class 1, 2 = Class 2, 3 = Class3.

## 4 Discussion

In this study, 31.4% of healthcare workers reported poor sleep quality, which is lower than the findings of a systematic evaluation of sleep disorders among Chinese healthcare workers before the COVID-19 epidemic (39.2%) (Qiu et al., 2020),but higher than the prevalence reported in the general population (Adams et al., 2017; Morin and Jarrin, 2022). However, the prevalence of sleep disturbances among Chinese healthcare workers varied greatly across studies, ranging from 12.9 to 78% (Feng and Yang, 2001; Lin et al., 2012), which may be related to the sample sizes, different PSQI cut-offs, and the regions surveyed. The results of a meta-analysis that included 31,749 participants showed that the pooled prevalence of sleep disturbances among healthcare professionals in northern China was significantly higher than in southern China (Qiu et al., 2020).In addition, the prevalence of sleep disturbances among healthcare workers in urban areas was higher than in rural/community areas, which may be attributed to the fact that healthcare institutions in urban areas are required to manage more emergencies, engage in shift work, and endure greater work pressure (Danet, 2021; Lucchini et al., 2020). However, the results of studies on regional disparities were not entirely consistent, as gender ratios, the timing of studies, different departments, and hospital grades all influenced the findings.

Using LPA, we grouped the sleep patterns of healthcare workers and identified three profiles: “good sleepers,” “inefficient sleepers,” and “poor sleepers.” “Good sleepers” and “inefficient sleepers” exhibited better overall sleep quality and reported very similar sleep problems, except for sleep efficiency. “Poor sleepers” reported lower overall sleep quality, characterized by more frequent sleep latency, shorter sleep duration, lower sleep efficiency and poorer subjective quality, as well as increased disturbances and daytime dysfunction. To the best of our knowledge, this is the first study to identify sleep patterns among Chinese healthcare workers. The largest proportion of healthcare workers were classified as “good sleepers,” while the smallest proportion were classified as “poor sleepers.” An Australian longitudinal study similarly identified the “good sleepers” pattern as “untroubled sleepers,” exhibiting few problems across all sleep dimensions, while the “poor sleepers” pattern was characterized as “troubled sleepers,” with more problems across all sleep dimensions (Leigh et al., 2015).Although previous literature has demonstrated the existence of sleep patterns characterized by various sleep problems, such as the “multiple problems” group (Yu et al., 2017), “sleep medication use” group (Mou et al., 2023), and “trouble falling asleep” group (Leigh et al., 2015), our findings suggest that sleep efficiency is a crucial dimension in differentiating sleep patterns. This is consistent with the findings of a UK study (Leng et al., 2014),which demonstrated that sleep duration is significantly less than time in bed. Sleep efficiency, commonly defined as the ratio of total sleep time to time in bed (TIB), plays a critical role in insomnia research and practice. Difficulty falling asleep is associated with psychological distress at bedtime, during attempts to fall asleep, and throughout normal waking hours, all of which perpetuate insomnia (Baglioni et al., 2010; Soehner and Harvey, 2012). Additionally, age is an important factor affecting sleep efficiency. A meta-analysis of objective studies involving 3,500 individuals aged 5 to 102 years showed that sleep efficiency in adults declines significantly with age (Ohayon et al., 2004).

The difference between good sleepers and inefficient sleepers may be related to irregular work routines (e.g., shift work), work-related stress, and the use of electronic devices, etc. Because shift work often includes night work, the normal sleep–wake cycle (circadian rhythm) is disrupted, resulting in potential consequences for shift workers’ sleep quality. A systematic review indicated that approximately 20–30% of shift workers experience significant symptoms of insomnia and excessive daytime sleepiness, which, in turn, leads to decreased sleep duration and sleep inefficiency (Booker et al., 2018). Clinical frontline healthcare workers often face exhausting daily demands and longer working hours; greater work pressure is also an important factor affecting sleep efficiency (Tucker et al., 2015).The use of electronic devices is becoming increasingly common, particularly for activities such as using devices in bed and browsing social media. While the overall quality of sleep can be better, there is a relative lack of sleep efficiency. A survey conducted during COVID-19 pandemic showed that 60.9% of healthcare workers experienced sleep disruptions or nightmares at least once per week due to device use (Stewart et al., 2021).

Regarding the characteristics of the population with different sleep patterns, the results of this study indicated that men had higher odds of being classified as “good sleepers,” while married individuals had higher odds of being classified as “inefficient sleepers.” These results are consistent with previous studies that have identified predictors of sleep in healthcare workers (Lv et al., 2023; Okechukwu et al., 2022; Zhou et al., 2024). Generally, the reasons women experience poorer sleep quality than men include behavioral factors (e.g., greater susceptibility to negative messages and sensitivity to emotions) and biological factors (e.g., hormonal regulation) (Cheng et al., 2020; Guidozzi, 2015). Married healthcare workers exhibited lower sleep efficiency, which may stem from their struggle to balance work and family obligations, leading to increased stress and sleep disturbances (Liu et al., 2020).

Regarding the relationship between sleep and psychiatric symptoms, this study found that psychological problems were more prevalent among “poor sleepers,” consistent with the findings of previous studies (Liu et al., 2022; Mou et al., 2023; Zhang et al., 2024). The relationship between sleep and psychiatric symptoms may be reciprocal (Scott et al., 2017). On one hand, individuals with shorter sleep duration may feel less rested and experience elevated stress levels (Kim and Lee, 2018). Perceived stress has been suggested as a risk factor for depressive symptoms (Racic et al., 2017). Additionally, physical and mental fatigue during the day, resulting from poor nighttime sleep, may disrupt circadian rhythms and cause hormonal changes that can contribute to psychological problems. Conversely, the increased risk of infections, excessive work patterns, coping with negative patient emotions, and frequent high arousal states are factors that can significantly affect sleep (Yin et al., 2020).

The results of this study provide practical insights into managing sleep health for healthcare workers. This study identified differences in the sleep patterns of healthcare workers, suggesting a need to develop targeted intervention strategies in the future. Specifically, in addition to focusing on poor sleepers, it is vital to target those who are good sleepers but exhibit lower efficiency. A meta-analysis of sleep interventions aimed at reducing psychiatric symptoms indicated that greater improvements in sleep quality aligned with greater enhancements in psychiatric status (Scott et al., 2017). Specific sleep management modalities include medication, multi-component cognitive-behavioral therapy for insomnia (CBTi), acupuncture, sleep hygiene education, and environmental adjustments, among other options. Healthcare institutions should also offer sleep-related training for healthcare workers. Both the Royal Society for Public Health (RSPH) and the Mental Health Foundation (MHF) recommend that primary healthcare training include awareness of and skills in assessing sleep problems (Mental Health Foundation, 2011). Hospital management should adjust shift work schedules based on the sleep quality, duration, and drowsiness levels of shift workers (Hulsegge et al., 2023). Trade union organizations in hospitals can organize cultural and recreational activities to help employees relax and improve their mood.

## 5 Limitation

This study has several limitations. First, the present study employed a cross-sectional design, which limited its ability to explore longitudinal sleep variability or identify causal relationships and potential mechanisms between sleep patterns and psychiatric symptoms. In the future, we will continue conducting annual sleep monitoring and analyze in-depth trends in the sleep patterns of healthcare workers by incorporating objective indicators (e.g., activity loggers). Second, certain socio-demographic variables (such as department, working hours, night shift, pregnancy, childbirth, etc.) were not captured in this study, these variables may positively predict sleep disorders. Third, as the sample was drawn from public hospitals, the findings may not be generalizable to all physicians in China, particularly those working in rural areas.

## 6 Conclusion

In this study, three sleep patterns were established using LPA among Chinese healthcare workers: (1) good sleepers (63.9%), (2) inefficient sleepers (30.3%), (3) poor sleepers (Class 3, 5.7%). The results of the multinomial logistic regression analysis indicated that gender and marital status were significant predictors of different sleep patterns. In comparison to “poor sleepers,” males exhibited higher odds of being classified as “good sleepers,” while married individuals were more likely to be categorized as “inefficient sleepers.” Significant differences in psychiatric symptom scores were observed across the three sleep patterns, with “poor sleepers” exhibiting the highest levels of psychiatric symptoms. Therefore, we recommend that hospital managers pay more attention to physicians classified as “poor sleepers” and “inefficient sleepers.” Targeted sleep management should be implemented to enhance sleep quality among healthcare workers and reduce their psychiatric symptoms.

## Data Availability

The raw data supporting the conclusions of this article will be made available by the authors, without undue reservation.
